# Eradication strategy for persistent methicillin-resistant *Staphylococcus aureus* infection in individuals with cystic fibrosis—the PMEP trial: study protocol for a randomized controlled trial

**DOI:** 10.1186/1745-6215-15-223

**Published:** 2014-06-12

**Authors:** Mark T Jennings, Michael P Boyle, David Weaver, Karen A Callahan, Elliott C Dasenbrook

**Affiliations:** 1 Johns Hopkins Medical Institutions, 1830 E. Monument Street, 5th floor, Baltimore, Maryland 21205, USA; 2 Case Western Reserve University School of Medicine, University Hospitals Case Medical Center and Rainbow Babies and Children’s Hospital, 11100 Euclid Avenue, Cleveland, Ohio 44106, USA

**Keywords:** Cystic fibrosis, MRSA, Randomized controlled trial, Vancomycin

## Abstract

**Background:**

The prevalence of methicillin-resistant *Staphylococcus aureus* (MRSA) respiratory infection in cystic fibrosis (CF) has increased dramatically over the last decade, and is now affecting approximately 25% of patients. Epidemiologic evidence suggests that persistent infection with MRSA results in an increased rate of decline in FEV_1_ and shortened survival. Currently, there are no conclusive studies demonstrating an effective and safe treatment protocol for persistent MRSA respiratory infection in CF.

**Methods/Design:**

The primary objective of this study is to evaluate the safety and efficacy of a 28-day course of vancomycin for inhalation in combination with oral antibiotics in eliminating MRSA from the respiratory tract of individuals with CF and persistent MRSA infection. This is a two-center, randomized, double-blind, comparator-controlled, parallel-group study with 1:1 assignment to either vancomycin for inhalation (250 mg twice a day) or taste-matched placebo for 28 days in individuals with cystic fibrosis. In addition, both groups will receive oral rifampin, a second oral antibiotic – trimethoprim/sulfamethoxazole (TMP/SMX) or doxycycline, protocol determined – mupirocin intranasal cream, and chlorhexidine body washes. Forty patients with persistent respiratory tract MRSA infection will be enrolled: 20 will be randomized to vancomycin for inhalation and 20 to a taste-matched placebo. The primary outcome will be the presence of MRSA in sputum respiratory tract cultures 1 month after the conclusion of treatment. Secondary outcomes include the efficacy of the intervention on: FEV_1_% predicted, patient reported outcomes, pulmonary exacerbations, and MRSA colony-forming units found in respiratory tract sample culture.

**Discussion:**

Results of this study will provide guidance to clinicians regarding the safety and effectiveness of a targeted eradication strategy for persistent MRSA infection in CF.

**Trial registration:**

This trial is registered at ClinicalTrials.gov (NCT01594827, received 05/07/2012) and is funded by the Cystic Fibrosis Foundation (Grants: PMEP10K1 and PMEP11K1).

## Background

Cystic fibrosis (CF) is the most common lethal autosomal recessive disorder in the Caucasian population [1]. With improvements in care, the average age of individuals living with CF continues to increase, with median predicted survival age now nearing 40 years [2]. One consequence of improving survival is the emergence of pulmonary infections with new and resistant pathogens. These infections are of importance because they may lead to respiratory failure, which continues to be the leading cause of mortality in individuals with CF. Methicillin-resistant *Staphylococcus aureus* (MRSA) is a particularly important emerging pathogen in CF. The prevalence of infection with MRSA in the CF community has increased from 4% in 1999 to 26.5% in 2012 [2].

Multiple observational studies utilizing the CF Foundation’s National Patient Registry database have demonstrated that infection with MRSA is associated with worse clinical outcomes [3-5]. Dasenbrook et al. demonstrated that persistent respiratory infection with MRSA is associated with a more rapid decline in lung function as measured by forced expiratory volume in one second (FEV_1_) percent predicted [3,4]. Sanders et al. suggested that MRSA is associated with failure to recover baseline lung function after a pulmonary exacerbation [5]. Finally, the hazard of death was significantly increased in CF patients with MRSA compared to CF patients without MRSA, even after adjustment for severity of illness.

Given the striking increase in both prevalence of MRSA infection in CF and evidence of a detrimental effect of MRSA on CF clinical outcomes, there has been growing interest in treatment protocols designed to treat and/or eradicate respiratory-tract MRSA in CF.

There have been several clinical studies in CF assessing MRSA treatment regimens and their effectiveness in eradicating MRSA from the respiratory tract. These studies have been limited by small study populations, lack of control groups, single-center retrospective design, variable follow-up, and failure to distinguish incident vs. persistent MRSA infection [6-9]. A retrospective review of 37 patients at an adult CF center in Manchester, UK, who had at least one positive MRSA culture and were treated with a combination of two oral antibiotics and nebulized vancomycin (200 mg four times a day for 5 days), reported that 81% of treated patients achieved MRSA eradication at 6 months [9]. A smaller study of seven adult CF patients with persistent MRSA found that five patients were culture-negative after completing a 6 month treatment of oral fusidic acid and rifampin [6]. Two studies that focused on the treatment of incident MRSA infection in CF reported eradication rates of 94% in 17 patients, using a combination of oral and IV antibiotics, and 55% in 15 patients treated with 5 days of oral and nebulized vancomycin (4 mg/kg/dose) [7,8].

There have been numerous reports of the clinical use of nebulized vancomycin in both CF and non-CF populations, all of which have demonstrated it to be well-tolerated and efficacious. The doses reported in those aged >12 years of age have included 125 mg twice a day, 250 mg four times a day, and 500 mg twice a day [10]. In the largest study to date, 51 non-CF patients received 125 mg of nebulized vancomycin twice a day for an average of 14.7 days to eradicate respiratory MRSA [11]. Eradication success rate was 84.3%. The authors reported that there were no adverse events associated with inhalation of vancomycin in the 51 participants. Additionally, a pilot study investigating the safety and tolerability of nebulized vancomycin in CF patients utilizing a single dose of 250 mg for inhalation in 10 patients found that it was safe and well tolerated [12]. All 10 patients tolerated the entire test dose without significant side effects, hypoxia, or evidence of sputum eosinophilia, and the mean relative change in FEV_1_ (L) 30 minutes after administration was −3.7% (range: −9.9% to +13.78%), demonstrating no significant bronchoconstrictive effect of vancomycin inhalation. Finally, there is significant clinical experience with the use of nebulized vancomycin in CF, as the CF treatment community frequently uses nebulized vancomycin in clinical care. A national compounding pharmacy which supplies nebulized vancomycin for clinical care to CF healthcare providers, reports treatment of over 95 CF patients with the dose described in the protocol without any reported treatment related serious adverse events [personal communication].

We are conducting a randomized controlled trial to determine if inhaled vancomycin for 28 days, in combination with an oral antibiotic regimen, can effectively eradicate MRSA from the respiratory tract of CF patients who are known to be persistently infected. We hypothesize that the use of such a strategy can result in MRSA clearance from the respiratory tract one month after completion of the protocol. The goal of this study is to determine if the 28-day combined inhaled vancomycin and oral antibiotic regimen is an effective and safe method of clearing persistent MRSA infection in CF.

## Methods/Design

This is a two-center, double-blind, comparator-controlled, randomized, and stratified by center and forced expiratory volume in one second percentage of predicted (FEV_1_%) (FEV_1_% ≤60% and FEV_1_% >60%), parallel-group study with 1:1 assignment to either vancomycin for inhalation (250 mg twice a day) or taste-matched placebo for 28 days in individuals with cystic fibrosis and chronic MRSA pulmonary infection. In addition, both groups will receive oral rifampin, a second oral antibiotic (TMP-SMX or doxycycline, protocol determined), mupirocin intranasal ointment, and chlorhexidine body washes. Forty patients with persistent respiratory tract MRSA infection will be enrolled: 20 will be randomized to vancomycin for inhalation and 20 to a taste-matched placebo.

All subjects will provide informed consent prior to enrollment in the study. Each subject will be administered study medication twice a day for 28 days. Subjects will be randomly assigned to the treatment or placebo. Evaluations will be taken at each of the six study visits. All subjects who receive at least one dose of study medication will be considered evaluable for safety and efficacy analyses. Incidence of adverse events will be monitored beginning at the time of consent. Efficacy assessments will be based on change in culture results, lung function, sputum MRSA density, cystic fibrosis quality of life questionnaire – revised (CFQ-R) scores, and number of exacerbations.

Total duration of subject participation will be 146 ± 9 days.

This study has been approved by the Institutional Review Boards at the Johns Hopkins University School of Medicine (Reference #: NA_00017536) and Case Western Reserve University School of Medicine (Reference #: 026513). Additionally, the U.S. Food and Drug Administration has granted an Investigational New Drug permit (IND # 114966) for sterile vancomycin 250 mg to be reconstituted in 5 mL sterile water and nebulized after reviewing this study protocol.

### Patient population

Subjects with a diagnosis of CF who meet the inclusion and exclusion criteria will be eligible for participation in this study. Inclusion and exclusion criteria are listed in Table 1. Criteria are designed to assure that only stable CF patients with persistent respiratory MRSA infection are studied.

**Table 1:** Study inclusion and exclusion criteria

**Inclusion criteria**	**Exclusion criteria**
● Male or female ≥12 years of age	● An acute upper or lower respiratory infection, pulmonary exacerbation, or change in routine therapy (including antibiotics) for pulmonary disease within 42 days of the Day 1 visit (2 weeks prior to screening visit)
● Confirmed diagnosis of CF based on the following criteria:	● Individuals on continuous inhaled antibiotics without interruption who are not willing to substitute vancomycin or placebo for their scheduled inhaled antibiotic during days 0–28 of the study
● 1. Positive sweat chloride >60 mEq/L (by pilocarpine iontophoresis) and/or	
● 2. A genotype with two identifiable mutations consistent with CF or abnormal nasal potential difference, and	
● 3. One or more clinical features consistent with the CF phenotype	
● Written informed consent (and assent when applicable) obtained from subject or subject’s legal representative and ability for subject to comply with the requirements of the study	● Use of oral or inhaled anti-MRSA drugs within two weeks of the screening visit
● Two positive MRSA respiratory cultures in the last 2 years at least 6 months apart, plus a positive MRSA respiratory culture at the screening visit and run-in (Day −14) visit	● History of intolerance to inhaled vancomycin or inhaled albuterol
● At least 50% of respiratory cultures from the time of the first MRSA culture (in the last 2 years) have been positive for MRSA	● History of intolerance to rifampin or both trimethorpim/sulfamethoxazole (TMP/SMX) and doxycycline
● FEV_1_ > 30% of predicted normal for age, gender, and height at screening	● Resistance to rifampin or both TMP/SMX and doxycycline at screening
● Females of childbearing potential must agree to practice an acceptable method of birth control (in the opinion of the investigator), including abstinence^a^. Female patients who utilize hormonal contraceptives as a birth control method must have used the same method for at least 3 months before study dosing	● Resistance to vancomycin at screening
	● Abnormal renal function, defined as creatinine clearance <50 mL/min using the Cockcroft-Gault equation for adults or Schwartz equation in children, at screening
	● Abnormal liver function, defined as ≥3x upper limit of normal, of serum aspartate transaminase or serum alanine transaminase, or known cirrhosis at the time of screening
	● Serum hematology or chemistry screening results which in the judgment of the investigator would interfere with completion of the study
	● History of or listed for solid organ or hematological transplantation
	● History of sputum culture with non-tuberculous *Mycobacteria* in the last 6 months
	● History of sputum culture with *Burkholderia Cepacia* in the last year
	● Planned continuous use of soft contact lenses while taking rifampin and no access to glasses
	● Current use of oral corticosteroids in doses exceeding the equivalent of 10 mg prednisone a day or 20 mg prednisone every other day
	● Taking voriconazole and unable to discontinue its use from run-in visit to Day 29 end-of-treatment visit
	● Administration of any investigational drug or device within 28 days of screening or within 6 half-lives of the investigational drug (whichever is longer)
	● Patients on inhaled antibiotics must have been on the same regimen for the 4 months prior to screening
	● Female patients of childbearing potential who are pregnant or lactating, or plan on becoming pregnant
	● Any serious or active medical or psychiatric illness, which in the opinion of the investigator, would interfere with patient treatment, assessment, or adherence to the protocol

a) Full details available in protocol.

### Trial intervention

Study participants will be randomized to receive nebulized vancomycin or taste-matched placebo. Nebulized vancomycin will be administered as 250 mg in 5 cc of sterile water, nebulized twice a day for 28 days. Placebo will consist of volume-matched (5 cc) and taste-matched (quinine 0.1 mg/mL) nebulized sterile water. Patients will use a Pari Sprint nebulizer and Pari Vios compressor as the delivery system, with a Pari Expiratory Filter to minimize environmental contamination with nebulized vancomycin. Patients will also be provided with an albuterol HFA MDI and instructed to take two puffs at home 5 to 60 minutes prior to study drug inhalation.

All study participants will receive oral rifampin (>45 kg: 600 mg daily; 35–35 kg: 450 mg daily; 25–34.9 kg: 300 mg daily), and oral trimethoprim/sulfamethoxazole (>45 kg: two DS tablets twice a day; 25–45 kg: one DS tablet twice a day). If a study participant is sulfa intolerant or their MRSA culture is trimethorpim/sulfamethoxazole (TMP/SMX) resistant, then they will receive oral doxycycline (>45 kg: 100 mg twice a day; 35–45 kg: 75 mg twice a day; 25–34.9 kg: 50 mg twice a day). Oral antibiotics will be administered for 28 days, in conjunction with the nebulized study drug.

To address potential nasal colonization with MRSA, study participants will be instructed to use mupirocin 2% intranasal ointment for the first 5 days of the treatment period: half of a single use tube applied into each nostril, twice a day. To address potential MRSA colonization of the skin, subjects will also be instructed to use Hibiclens 15 cc liquid skin cleanser packets (4% chlorhexidine gluconate) once weekly in the shower for 4 weeks to minimize body colonization. Finally, to address potential home sources of MRSA, participants will wipe down all high-touch home surfaces with Sani-Cloth Alcohol Free Germicidal wipes and wash bed linen and towels in hot water weekly for the first three weeks of the study. See Table 2 for a list of study interventions.

**Table 2:** Study interventions

**Intervention arm (n = 20)**	**Placebo arm (n = 20)**	**All study participants (Intervention + Placebo arms)**		
● Vancomycin: 250 mg/5 cc sterile water nebulized two times a day, Days 1–28	● Placebo: quinine 0.5 mg/5 cc sterile water nebulized two times a day, Days 1–28	● Oral Rifampin: Days 1–28		
		● 1. >45 kg: 600 mg by mouth daily		
		● 2. 35–45 kg: 450 mg by mouth daily		
		● 3. 25–34.9 kg: 300 mg by mouth daily		
		● Oral TMP/SMX^a^ (DS-160/800): Days 1–28	OR	● Oral Doxycycline^b^: Days 1–28
		● 1. >45 kg: Two DS tabs by mouth twice a day		● 1. >45 kg: 100 mg by mouth twice a day
		● 2. 25–45 kg: One DS tab by mouth twice a day		● 1. 35–45 kg: 75 mg by mouth twice a day
				● 2. 35–45 kg: 75 mg by mouth twice a day
		● Mupirocin 2% intranasal ointment: apply to each nasal cavity twice a day for Days 1–5 of study		
		● Hibiclens (4% chlorhexidine gluconate) liquid skin cleanser: use three packets once weekly in the shower, Days 1–28		
		● Wipe down of high touch household surfaces with Sani-Cloth Alcohol Free Germicidal wipes: use once a week, Days 1–28		
		● Wash all linen and towels in hot water: weekly, Days 1–21		

^a^Participants with MRSA susceptible by MIC testing to TMP/SMX and without history of sulfa allergy will be treated with trimethoprim/sulfamethoxazole.

^b^Participants with MRSA resistant by MIC testing to TMP/SMX and without history of doxycycline allergy will be treated with doxycycline.

All of the participant’s usual CF medications and treatments are allowed except non-study anti-MRSA antibiotics beginning 2 weeks prior to the screening visit and throughout the study (unless medically indicated). Subjects should remain on a stable medical regimen throughout the entire study period, with the exception that patients on continuous inhaled antibiotics will stop their non-study drug inhaled antibiotic during the 28 day treatment period. If subjects are on an alternating 28-day “on”/”off” cycle of an inhaled antibiotic, study drug dosing will occur during the “off” cycle. There will be no introduction of other new chronic therapies during the study period unless medically required.

### Randomization

Up to 40 eligible patients will be enrolled and randomly assigned to vancomycin for inhalation or placebo treatment in a 1:1 ratio using a SAS-based, computer-generated randomization scheme. The randomization will be performed in blocks of random sizes and stratified by FEV_1_% and center. The randomization will also be designed to assure an equal division between placebo and treatment among the first 16 participants in preparation for the first data and safety monitoring board (DSMB) safety review (after 16 participants are enrolled). After the initial DSMB safety review and approval, eligible subjects aged 12–17 years may be enrolled.

### Blinding

The identity of test and control treatments will not be known to investigators, research staff, or patients. The following study procedures will be in place to ensure double-blind administration of study treatments:

• Access to the randomization code will be strictly controlled. Access to the randomization code will be limited to the randomization programmer, manager of the data management unit as a backup to the randomization programmer, and the drug packaging group.

• Packaging and labeling of test and control treatments will be identical.

• Measured serum and sputum vancomycin levels will not be available to the investigators until data base lock.

• The study blind will be broken on completion of the clinical study and after all adverse events have been evaluated for relationship to study drug, coded, and reviewed by the DSMB.

• During the study, the blind may be broken only in emergencies when knowledge of the patient’s treatment group is necessary for further patient management.

### Participant follow-up

Study participants will be followed for up to 155 days. The duration of subject participation will include: Screening/Run-in: up to 30 days; Treatment: 28 days; Primary Endpoint: Day 58; Follow-up: 3 months after completion of 28-day treatment period. See Table 3 for a complete list of study visits, events, and study procedures by event.

**Table 3:** Schedule of study visits

	**Screening visit (Day-28) (±2 days)**	**Run-in visit (Day −14) (±4 days)**	**Visit 1 (START TREATMENT) (Day 1)**	**Visit 2 (REPEAT ADMIN) (Day 2–7)**	**Phone visit 1 (Day 7–14)**	**Visit (MID-POINT TREATMENT) (Day 14) (±2 days)**	**Phone visits 2 & 3 (Day 14–21) (Day 21–28)**	**Visit 4 (END TREATMENT) (Day 29) (±1 day)**	**Visit 5 1 month monitoring (Day 58) (±4 days)**	**Visit 6 3 month monitoring (Day 118) (±7 days)**	**Early withdraw visit**
Informed consent	**X**										
Randomization		**X**^**n**^									
Medical history (a)	**X**^**a**^	**X**	**X**	**X**		**X**		**X**	**X**	**X**	**X**
Demographics	**X**										
Complete physical exam	**X**							**X**		**X**	**X**
Abbreviated physical exam		**X**	**X**	**X**		**X**			**X**		
Height, weight	**X**	**X**	**X**	**X**		**X**		**X**	**X**	**X**	**X**
Vital signs, oximetry (b)	**X**	**X**	**X**^**b**^	**X**^**b**^		**X**^**b**^		**X**	**X**	**X**	**X**
CFQ-R (c)			**X**			**X**		**X**	**X**	**X**	**X**
Administer albuterol (d)	**X**	**X**	**X**	**X**		**X**		**X**	**X**	**X**	**X**
Spirometry (e)	**X**	**X**	**X**^**e**^	**X**^**e**^		**X**		**X**	**X**	**X**	**X**
Sputum induction procedure (f)	**X**	**X**				**X**		**X**	**X**	**X**	**X**
Sputum culture and sensitivity	**X**	**X**				**X**		**X**	**X**	**X**	**X**
Sputum MRSA colony forming units (g)	**X**	**X**				**X**		**X**	**X**		**X**^**o**^
Small colony variants of MRSA (g)	**X**	**X**				**X**		**X**	**X**	**X**	**X**
MRSA: comprehensive genetic strain analysis (g)	**X**	**X**				**X**		**X**	**X**	**X**	**X**
Sputum cell count (h)		**X**				**X**		**X**	**X**		
Sputum cytokine measurements (h)		**X**						**X**			
Nasal, axillary, rectal swabs for culture (i)		**X**^**i**^						**X**^**i**^	**X**^**i**^		
Administration of study drug			**X**^**b**^	**X**^**b**^		**X**^**b**^					
Expectorated sputum vancomycin level (j)			**X**								
Serum vancomycin levels (k)			**X**^**k**^			**X**^**k**^					**X**^**q**^
Chemistry/LFTs/hematology/CRP	**X**		**X**			**X**		**X**	**X**		**X**^**p**^
Pregnancy test (Urine or Serum) (l)	**X**^**l**^		**X**^**l**^			**X**^**l**^					
Initiate subject diary		**X**									
Subject diary review			**X**	**X**	**X**	**X**	**X**	**X**	**X**		**X**^**p**^
Concomitant medication review	**X**	**X**	**X**	**X**	**X**	**X**	**X**	**X**	**X**	**X**	**X**
Adverse experiences review		**X**	**X**	**X**	**X**	**X**	**X**	**X**	**X**	**X**	**X**
Hibiclens test hand washing (m)		**X**									
Provide snack			**X**			**X**					
Administration of rifampin, protocol determined oral antibiotic, nasal mupirocin and Hibiclens			**X**			**X**					
Dispense supply of study drug, nebulizer, rifampin, protocol determined oral antibiotic, mupirocin and Hibiclens and provide teaching			**X**			**X**					
Collect used and unused study drug and containers						**X**		**X**			**X**^**p**^

^a^Spontaneous expectorated sputum collected 5 ± 4 minutes after nebulizing study drug. Gargle with 30 cc of saline for ten seconds x3 prior to collection.

^b^Peak drawn 60 ± 10 minutes after end of nebulization on day 1; trough drawn with other blood tests prior to nebulization on day 14.

^c^Serum pregnancy test done at screening, urine at other time points.

^d^Wash with water and one packet of Hibiclens hand cleanser for 2 minutes. Skin reaction will be observed for 15 minutes after washing.

^e^Randomization will be a non-visit task on Day −4 (±3 days) once culture results from run-in visit confirm presence of MRSA.

^f^CFU’s will be done if early withdrawal visit occurs between visit 2 and visit 4.

^g^If applicable, only if early withdrawal visit occurs between visit 1 and visit 4.

^h^If applicable, only if early withdrawal visit occurs before visit 3.

^i^History of previous treatment for MRSA, number of MRSA cultures, and length of MRSA positivity at screening visit.

^g^Continuous pulse oximetry is performed during nebulization of study drug and for 5 minutes afterward on Days 1, Repeat admin visit and Day 14. Observe for 1 h post-treatment.

^k^On days CFQ-R is performed, it is done before any other interventions.

^l^Two puffs of Albuterol HFA 10–30 minutes prior to spirometry and 10–60 minutes prior to study drug nebulization on Days 1 and 14.

^m^Single spirometry value also obtained immediately after and 15 minutes after study drug nebulization on Day 1 and Repeat Administration Day.

^n^Follow TDN Sputum Induction protocol 517.01; throat swab if inadequate sputum produced.

^o^Only if culture grows MRSA.

^p^Follow TDN Sputum Processing Protocol 508.01.

^q^Rectal swabs optional and only age ≥18; no rectal swab on day 58.

### Outcome measures

The primary outcome of this study is the percentage of patients MRSA free by induced sputum respiratory tract culture 1 month (Visit 5, Day 58) after completion of 4-week eradication protocol in intervention arm vs. control arm.

Secondary efficacy outcomes include: i) percentage of patients MRSA free at visit 4 (Day 29) and Visit 6 (Day 118); ii) change in FEV_1_ from baseline (Day 1) at visit 4 (Day 29), Visit 5 (Day 58) and visit 6 (Day 118); iii) change in FEV_1_ from screening (Day −28) at Visit 4 (Day 29), visit 5 (Day 58) and visit 6 (Day 118); iv) time to first pulmonary exacerbation, measured from Day 1; v) total number of exacerbations at visit 5 (Day 58) and visit 6 (Day 118); vi) change in patient reported CFQ-R (respiratory) from baseline (visit 1) to visit 3 (Day 14), visit 4 (Day 29), visit 5 (Day 58), and visit 6 (Day 118); 7) number of patients with newly developed MRSA resistance to vancomycin, TMP/SMX, doxycycline, and/or rifampin at any point during follow-up.

Secondary safety outcomes include: i) chemistry panel, aspartate transaminase, alanine transaminase, alkaline phosphatase, and complete blood count with differential, at screening (Day −28), visit 3 (Day 14), visit 4 (Day 29), and visit 5 (Day 58); ii) incidence of adverse events; iii) continuous oxygen saturation throughout first nebulized vancomycin dose; iv) spirometry checked immediately and 15 min (±10 min) after first nebulized vancomycin dose; v) hand washing with chlorhexidine body wash at visit 1 (Day 1) to monitor for skin sensitivity.

Additional secondary outcomes include: i) serum vancomycin peak level 60 minutes after completion of first dose of inhaled vancomycin at visit 1; ii) trough serum vancomycin level to be drawn prior to nebulization of vancomycin at visit 3 (Day 14 – 2 weeks into nebulized vancomycin); iii) sputum vancomycin level at visit 3 (Day 14), 30 minutes after nebulizing vancomycin in patients; iv) change in minimum inhibitory concentration (MIC) of vancomycin, rifampin, TMP/SMX, and tetracycline for MRSA. Centers for Disease Control and Prevention guidelines classify MRSA as vancomycin-intermediate *Staphylococcus aureus* if the MIC for vancomycin is 4–8 μg/mL, and classify MRSA as vancomycin-resistant *Staphylococcus aureus* if the vancomycin MIC is ≥16 μg/mL. We will also monitor for those MIC’s that are initially less than 2 μg/mL and convert to 2 μg/mL; 5) rectal swabs at run-in visit (Day −14), visit 4 (Day 29), and visit 5 (Day 58) to screen for the development of vancomycin-resistant enterococcus.

### Statistical analysis

The following demographic variables at screening will be summarized by treatment arm: race, gender, age, height, and weight. All eligible patients who are randomized into the study and receive at least one dose of the study drug (the Safety Population) will be included in the safety analysis.

Our hypothesis for our primary outcome states that the treatment arm will result in significantly greater eradication of persistent MRSA from the respiratory tract of CF adolescents and adults compared to the placebo arm on day 58. Our primary analysis will include comparing the proportion of CF patients in the treatment arm who have a negative MRSA culture at visit 5 to the proportion of patients in the placebo arm who have a negative MRSA culture at visit 5. Secondary analyses will include the above comparisons, but at visits 4 and 6. χ^2^ or Fishers exact test will be used to determine statistical significance. A secondary analysis will also be performed comparing proportions of MRSA positive cultures among the three cultures obtained at visits 3, 4, and 5 during the study. Generalized estimating equations (GEE), using a logit link, will be used to model this longitudinal data. The GEE model will be used to test for main effects of treatment arm, time, and treatment × time interaction. Furthermore, sensitivity analyses will be performed to evaluate the robustness of the primary outcome to missing culture data (missing data should be minimal) using i) imputation with last observation carried forward and multivariate imputation by chained equations, ii) treating all missing cultures as negative, and iii) treating all missing cultures as positive. Finally, the statistical analyses, evaluating both primary and secondary outcomes, will be adjusted for the presence of *Pseudomonas* positive sputum cultures, as well as for the concurrent use of azithromycin during the course of the study period.

Our hypothesis for the secondary outcomes states that the treatment arm will improve clinical measures (lung function, quality of life, and exacerbations) compared to placebo. Statistical significance for differences in lung function (FEV_1_%) at visit 4 will be assessed between the two treatments using a 2-sample *t*-test. We will use linear mixed models to longitudinally analyze and compare the change in lung function over 155 days in the two arms. CFQ-R respiratory scores will be expressed as mean ± standard deviation and statistical significance assessed between the two arms at visit 4 using a 2-sample *t*-test. A Kaplan-Meier plot will be used to graphically display estimates of the survivor function for the proportion of patients who do not have an exacerbation through day 118. Hazard ratios and 95% confidence intervals due to treatment will be calculated using a Cox proportional hazards regression model. Pre-specified potential variables that may be adjusted for include gender, season of enrollment, and center.

Our third hypothesis is that the treatment will have similar effects with regards to the development of antimicrobial resistance and adverse events compared to placebo. There has been concern in previous methicillin-sensitive *Staphylococcus aureus* treatment studies of an increased risk of gram-negative pathogen acquisition; therefore, we will monitor for this development [13]. Rates of occurrence of development of microbial resistance, gram-negative pathogens will be summarized separately by treatment arm. Between-group comparisons will be made using a χ^2^ test or Fishers exact test.

Adverse event rates will be coded by body system and MedDra classification term. Adverse events will be tabulated by treatment group and will include the number of patients for whom the event occurred, the rate of occurrence, and the severity and relationship to study drug. Fisher’s exact test will be used to compare the various treatment groups with respect to incidence of the more commonly occurring adverse events.

No interim analyses are planned. A safety analysis will be planned after the first 16 adults are enrolled. If the DSMB agrees based on initial safety data, enrollment eligibility will then be expanded to include those aged 12–17 as well.

### Sample size

This study is powered on the primary outcome: the percentage of patients that are induced sputum negative for MRSA at visit 5 (day 58). We are planning a study with 20 experimental and 20 control patients. There are no treatment studies published in CF patients with persistent MRSA to guide the assumptions for the primary outcome for the two arms. In fact, the results from this study will be used to power future multi-center trials. Based on our experience, treatment with oral antibiotics in those with persistent MRSA results in an approximate eradication rate of 15–25%. An experimental arm eradication rate of 75% would be considered significant, though some may even consider a lower percentage significant as well. Based on these assumptions, we have greater than 80% power if the MRSA eradication rate in the experimental arm is at least 65%, assuming a 20% eradication rate in the placebo arm.

## Discussion

MRSA pulmonary infection is a clinically significant complication for individuals with cystic fibrosis. The dramatic increase in the prevalence of MRSA infection in CF and the new understanding that persistent infection with this resistant pathogen increases the rate of decline of lung function and shortens survival for CF patients has made finding an effective and safe treatment approach an urgent need. Unfortunately, there is currently no clear data as how to best treat persistent MRSA infection in CF. This trial will evaluate the efficacy and safety of a particularly promising treatment protocol utilizing inhaled vancomycin and multiple oral antibiotics to eradicate MRSA from CF patients with known persistent MRSA infection. Results of this trial will provide guidance both for CF clinicians and for future investigative efforts directed at this increasingly important challenge in CF.

## Trial status

The first patient was enrolled in October 2012. Recruitment is ongoing.

## Abbreviations

CF: Cystic fibrosis; CFQ-R: Cystic fibrosis quality of life questionnaire – revised; DSMB: Data and safety monitoring board; FEV_1_: Forced expiratory volume in one second; FEV_1_%: Forced expiratory volume in one second percentage of predicted; GEE: Generalized estimating equations; MIC: Minimum inhibitory concentration; MRSA: Methicillin-resistant *Staphylococcus aureus*; TMP/SMX: Trimethorpim/sulfamethoxazole (Bactrim).

## Competing interests

The authors declare that they have no competing interests.

## Authors’ contributions

MTJ wrote the manuscript and helped in performance of the study. MPB designed the protocol, helped in performance of the study, and contributed to the manuscript. DW and KAC helped in design of the protocol and contributed to the manuscript. ECD designed the protocol, helped in performance of the study, and contributed to the manuscript. All authors read and approved the final manuscript.
